# Rapid serological detection of smooth *Brucella* strain antibodies in bovine and sheep using a dynamic flow immunochromatographic test

**DOI:** 10.3389/fcimb.2026.1804374

**Published:** 2026-06-04

**Authors:** Kaiyutai Zhou, Chenye You, Fucheng Wang, Lulu Li, Liyan Lian, Yuanzheng Jia, Xiangan Han, Wei Jiang

**Affiliations:** 1 State Key Laboratory of Dairy Biotechnology, Shanghai Engineering Research Center of Dairy Biotechnology, Dairy Research Institute, Bright Dairy & Food Co., Ltd, Shanghai, China; 2 Shanghai Veterinary Research Institute, Chinese Academy of Agricultural Sciences, Shanghai, China

**Keywords:** bovine, brucellosis, dynamic flow immunochromatographic test, lipopolysaccharides, sheep, smooth lipopolysaccharide

## Abstract

Brucellosis, a severe zoonotic disease with over 2,000,000 annual human cases, poses a major threat to the livestock industry and public health. Early and accurate diagnosis in animals is crucial for control, but current methods are often time-consuming, equipment-dependent, or prone to cross-reactivity. This study developed a cost-efficient dynamic flow immunochromatography (DFICT) technique for the accurate diagnosis of bovine and sheep Smooth brucellosis. The assay utilizes a test strip with Smooth lipopolysaccharide and *Streptococcus* protein G (SPG) immobilized as the Test (T) and Control (C) lines, employing gold nanoparticle-SPG conjugates as the tracer. To perform the DFICT, 100 μL of the liquid conjugate is applied to the reagent well, followed by 5 μL of serum to the sample well. The results were observable within 10 min by the naked eye. The DFICT demonstrated high sensitivity, detecting antibodies in sera diluted up to 1:64, and exceptional specificity with no cross-reactivity to other common pathogens. The DFICT can be stored for 12 months at 4 °C with no loss of sensitivity or specificity. Clinical validation with 80 bovine and 161 sheep field sera showed strong agreement with standard methods (ELISA, RBT), achieving sensitivities of 94.6% (bovine) and 98.2% (sheep), and specificities of 100% and 84.0%, respectively. It’s simple, equipment-free operation and reliability make the established DFICT a vital and necessary tool for large-scale, on-site screening, significantly enhancing Smooth brucellosis surveillance and control capabilities.

## 1 Introduction

Brucellosis, a widespread zoonotic disease caused by *Brucella* species, primarily affects livestock such as bovine, sheep, and goats, posing significant risks to both public health and the agricultural economy (Liu et al., 2024). With over 2,000,000 human cases reported annually worldwide (Holloway et al., 2025), its transmission occurs mainly through contact with infected animals or consumption of contaminated products (Wei et al., 2025).The principal clinical impact of *Brucella* infection is concentrated in the reproductive tract. Infected females commonly present with late-term abortion, retained fetal membranes, and mammary gland colonization. Mammary colonization can lead to persistent bacterial shedding in milk, thereby contaminating the environment and facilitating horizontal transmission to neonates and herd mates (Ghssein et al., 2025; Holloway et al., 2025). In males, orchitis and epididymitis are common and are associated with reduced semen quality and, in severe cases, infertility (Kimera et al., 2025). In some regions, maintaining mixed herds of bovine, sheep, and goats is common practice, thereby increasing the risk of interspecies transmission of brucellosis (Xingxing et al., 2024). Human infection can lead to severe complications including arthritis, meningitis, and neurological disorders, highlighting the critical need for effective control strategies (Qureshi et al., 2023). While the expanding livestock sector meets growing demand for animal products, it has also increased animal movement, thereby facilitating brucellosis spread and threatening both the economy and public health (Khurana et al., 2021).The primary transmission route is through contaminated food, with under-diagnosis in endemic regions further impeding control (Dadar et al., 2021). Thus, the development of an effective and unified test for the early and accurate diagnosis of infected animals, particularly one applicable to both bovine and sheep, is vital to prevent the spread of *Brucella* infection.

Current diagnostic methods, including bacterial culture, serological assays, and molecular techniques, present limitations (Dadar et al., 2021). While bacterial culture is the gold standard, it requires Biosafety level 3 facilities, is time-consuming, and is impractical for large-scale use (McGiven, 2013; Prakash et al., 2021). Serological tests, such as the Rose Bengal plate agglutination test (RBT), complement fixation test (CFT), and serum tube agglutination test (SAT), are widely used alternatives. Nevertheless, they are susceptible to non-specific cross-reactions with other bacteria, leading to false-positive results (Dawood et al., 2023). Moreover, the interpretation of results can be subjective, potentially resulting in misdiagnosis or underdiagnosis (Fang et al., 2021). Consequently, to achieve acceptable diagnostic accuracy, these methods are frequently used in combination, a practice that significantly extends the testing timeline. In recent years, advanced molecular and serological assays, including enzyme-linked immunosorbent assay (ELISA), polymerase chain reaction (PCR), and quantitative real-time PCR (RT-qPCR), have improved early detection rates by offering high specificity and sensitivity (Thakur et al., 2018; Yagupsky et al., 2019). Despite their superior accuracy, these techniques necessitate expensive instrumentation, highly trained personnel, and are relatively time-consuming, which collectively limits their utility for rapid field screening (Zhang et al., 2022).

To address these diagnostic limitations, the immunochromatographic test (ICT) offers a promising point-of-care platform. Recognized for its operational simplicity, cost-effectiveness, and rapid results, ICT is particularly suited for on-site brucellosis screening (Novak et al., 2022), and its application is well-documented (Kakkar et al., 2024). Specific developments include a colloidal gold strip for detecting smooth *Brucella* (Wu et al., 2024a) and a colored latex microsphere strip for differentiating *B. abortus* (Shi et al., 2022). It is noteworthy that most existing ICTs for animal brucellosis are typically designed for single-species detection, which limits their diagnostic efficiency in mixed farming systems. Moreover, despite these technical advances, conventional ICTs continue to face challenges related to manufacturing complexity, sample volume requirements, and production costs—factors that remain critical barriers to their large-scale field deployment.

Building on our previous development of a dynamic flow immunochromatographic test (DFICT) that integrates immunochromatography with fluid dynamics (Jiang et al., 2015) this study adapts the platform for brucellosis detection in bovine and sheep. The DFICT system utilizes a liquid gold conjugate, which eliminates the need for drying facilities or strict humidity control during manufacturing. This key advantage significantly streamlines the production process, reduces costs, and makes the technology particularly suitable for use in resource-limited settings. Additional benefits include minimal sample volume requirements (as low as 5 μL), high selectivity, and operational efficiency—collectively overcoming major limitations of conventional immunochromatographic tests. Smooth lipopolysaccharide is a key virulence-associated surface component of *Brucella melitensis*. Anti-LPS antibodies can appear early after infection and may persist throughout the course of disease. In this study, high-purity S-LPS was isolated and used as the capture antigen to bind anti–S-LPS antibodies, thereby providing a reliable early serological target for the DFICT system. Here, we evaluate the performance of DFICT using clinical serum samples through sensitivity and specificity testing, along with comparative validation against commercial ELISA and RBT methods. By providing a unified, reliable, and field-friendly detection solution for both bovine and sheep, this approach supports broader application in integrated surveillance of mixed livestock populations.

## 2 Materials and methods

### 2.1 Serum samples and reagents

All these control serum samples used in this study were obtained from our laboratory’s repository. This including 3 standard *Brucella*-positive sheep sera, 2 standard *Brucella*-positive bovine sera, and 10 standard negative sera (5 from sheep and 5 from bovine, all sourced from healthy animals). Additionally, positive control serum samples for common pathogens in sheep and bovine—including *Escherichia coli* O157, *Salmonella dublin*, *Yersinia enterocolitica*, *Staphylococcus aureus*, foot-and-mouth disease virus (FMDV), and *Pasteurella multocida*—were prepared in our laboratory. Furthermore, we collected 161 sheep and 80 bovine serum samples from various farms in China, where animals had either been immunized or not immunized with the *Brucella* vaccine. Vaccinated sheep received a single subcutaneous dose of *B. melitensis* M5 vaccine, while cattle were immunized with *B. abortu* A19 strain. All serum samples were stored at -80 °C until use.

Nitrocellulose (NC) membranes, glass fiber membranes, absorbent pads, and PVC sheets were purchased from Millipore Corporation (Shanghai, China). Affinity-purified rabbit anti-donkey IgG whole molecule was obtained from Zhaorui Biotech Co., Ltd (Shanghai, China). Hydrogen tetrachloroaurate hydrate (HAuCl4), trisodium citrate, bovine serum albumin (BSA), and sodium azide were purchased from Sigma Chemical Company (USA). Proteinase K, RNase A, and deoxyribonuclease I (DNase I) were purchased from Solarbio Technology Co., Ltd. (Beijing, China). SPG was acquired from Yuanye Bio-Technology Co., Ltd (Shanghai, China). The *Brucella* antigen for the Rose-Bengal plate agglutination test was purchased from Qingdao Lijian Bio-Tech Co., Ltd (Shandong, China). The *Brucella* antibody competitive ELISA (cELISA) kit was purchased from Eurofins Technologies Ingenasa (Spain). All solvents, chemicals, and salts used were of analytical grade, and solutions were prepared using Milli-Q18 Ω water from a Millipore Purification System.

### 2.2 Preparation of immunoassay reagents

Lipopolysaccharide (LPS) was extracted from *Brucella abortus* S2308 using a hot phenol-water extraction. Briefly, 5 g of wet-weight bacteria were resuspended in hot distilled water (65 °C). An equal volume of 90% phenol solution was added, and the mixture was stirred continuously at 65 °C for 15 min. After cooling, the mixture was centrifuged at 10,000 × *g* for 15 min at 4 °C. The aqueous phase containing LPS was recovered by filtration to remove cellular debris. The phenol phase (bottom layer) was also filtered. Subsequently, 1% saturated sodium acetate-methanol solution (three times the volume of the LPS-containing solution) was added, and the mixture was allowed to precipitate for 2 h at 4 °C. The precipitate was collected by centrifugation at 10,000 × *g* for 10 min, suspended in sterilized water, and centrifuged again; the supernatant was retained.

After additional centrifugation, the supernatant was dialyzed to obtain a crude LPS extract. For further purification, crude LPS was treated with DNase I, RNase A, and proteinase K (20 µg/mL each) at 37 °C for 2 h to remove nucleic acid and protein contaminants. The digested solution was then dialyzed against deionized water to obtain purified LPS.

LPS purity was assessed by sodium dodecyl sulfate-polyacrylamide gel electrophoresis (SDS-PAGE) with silver staining. To check for protein contamination, gels were stained with Coomassie Brilliant Blue R250 and decolorized; lanes were examined for any protein bands.

### 2.3 Preparation of gold-SPG conjugates

Colloidal gold particles were prepared following a previously described method with minor modifications (Guo et al., 2015). All glassware was cleaned with a potassium dichromate-sulfate acid solution and rinsed with ultrapure water. A 100 mL solution of 0.01% HAuCl_4_ was heated to boiling with magnetic stirring. Then, 2 mL of 1% trisodium citrate solution (w/v) was quickly added under continuous stirring. The solution color changed from dark gray to red; after stabilization, heating continued for 5 min before cooling to room temperature (RT). The pH was adjusted to 6.0 using 0.2 M K_2_CO_3_. Next, 1 mL SPG (1.0 mg/mL) was slowly added to the 100 mL colloidal gold solution and reacted at RT for 30 min with stirring. To stabilize the gold particles, 100 μL of 10% BSA was added to achieve a final concentration of 1% (w/v). The mixture was centrifuged at 10,000 rpm for 30 min at 4 °C, and the supernatant was discarded. The pellet was suspended in 0.01M PBST containing 1% (w/v) BSA, 0.9% (w/v) NaCl, and 0.05% (w/v) sodium azide, and stored at 4 °C.

### 2.4 Preparation of the DFICT strip

**Figure 1:**
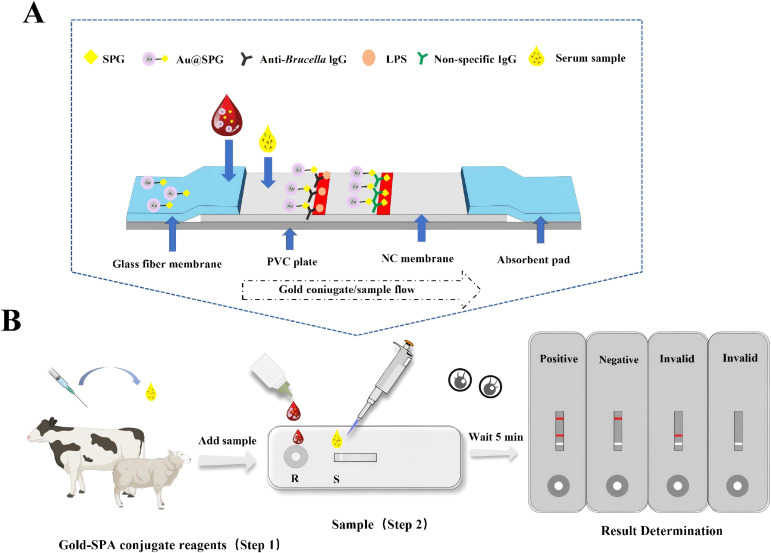
**(A)** Schematic diagram of the DFICT strip procedure for detecting brucellosis in cattle and sheep. **(B)** Structure and detection principle of the DFICT strips.

The DFICT test strip utilize a liquid gold conjugate reagent that binds to specific antibodies during sample migration, eliminating the need for conjugate and sample pads used in traditional immunochromatographic test (ICT) strips. The assembly process is illustrated in **Figure 1A**. A plastic-backed card was sequentially assembled with an NC membrane, a glass fiber membrane, and an absorbent pad, with each component overlapping the next by 1–2 mm to ensure consistent fluid flow. *Brucella* LPS and SPG were immobilized on the NC membrane as the test line (T-line) and control line (C-line), respectively, using a Quanti 3000 Biojets sprayer connected to an XYZ Biostrip Dispenser (Bio-Dot, Irvine, CA, USA). The spray volume was 1 μL/cm, with 4 mm between the two lines. After drying at 37 °C for 3 h, the strips were cut into 4 mm widths using Bio-Dot CM4000 Cutter (Irvine, CA, USA). The strips were then sealed in aluminum pouches with desiccant and stored at RT. The liquid gold conjugate reagent was stored at 4 °C.

### 2.5 Evaluation of the sensitivity, specificity, and stability of the DFICT

The sensitivity of the DFICT strips was evaluated using serial dilutions (from 1:2 to 1:64 in 0.01 M PBS, pH 7.2) of standard *Brucella*-positive sheep and bovine sera. Serum samples from healthy sheep and bovine served as negative controls. For testing, 5 μL of each serum sample was applied. This experiment was performed in triplicate.

To assess specificity, standard *Brucella*-positive sheep and bovine sera were used as positive controls, alongside positive serum samples for other pathogens (e.g., *E. coli* O157, *Y. enterocolitica*, *S. dublin*, and FMDV, *P. multocida*). Each sample group was tested three times.

To determine stability, test strips and conjugate reagents were stored at both room temperature (RT) and 4 °C for 3, 6, 9, and 12 months. After each storage period, sensitivity and specificity were re-evaluated using the standard positive and negative sera.

### 2.6 Clinical evaluation of the DFICT compared to traditional methods

A total of 161 sheep and 80 bovine field serum samples were tested using the DFICT, and the results were compared with those from reference methods the RBT, iELISA, and cELISA. The operational procedures for these methods are summarized below:

RBT: The test was performed using a commercial kit per the manufacturer’s instructions. Briefly, 30 μL of serum was mixed with 30 μL of antigen. Results were read within 4 minutes, with any agglutination considered positive.

iELISA: The plate was coated with LPS, washed, and stored at -20 °C. With all reagents at room temperature, controls and test sera were added and incubated for 1 hour. After washing, enzyme-conjugated antibody was added, followed by another 1-hour incubation and wash. The reaction was developed using TMB substrate, stopped, and the OD_450_ was measured. The test was valid if the positive control OD_450_ was ≥1.0 and the negative control was <0.2. Samples with an S/N ratio (Sample OD_450_/Negative Control OD_450_ × 100%) ≥ 2.1 were considered positive.

cELISA: The procedure was carried out using a commercial cELISA kit according to the protocol. All reagents were equilibrated to room temperature first. Then, 100 μL of controls and test sera were added, and the plate was incubated for 1 hour. After four washes, 100 μL of conjugate was added, and the plate was incubated for another hour. Following four washes, 100 μL of TMB substrate was added for a 10-minute development, then the reaction was stopped, and the OD_450_ was measured within 5 minutes. The test was valid if the positive control OD_450_ was <0.35 and the negative control was >1.0. The Percent Inhibition (PI) was calculated as PI = [1 – (Sample OD_450_/Negative Control OD_450_)] × 100%, and samples with PI ≥ 40% were considered positive.

DFICT: A100 μL aliquot (approximately 2 drops) of the gold-SPG conjugate reagent was added to the reagent well. Results were read visually within 5 min: the appearance of both T and C lines indicated a positive result, while only a C line indicated a negative result. Each test strip was imaged with a smartphone 5 min after sample application. Images were uploaded to a computer and analyzed using ImageJ 1.53 software to generate a contour intensity map. The signal intensities of the T line and C lines were quantified, and the T/C ratio (T line intensity divided by C intensity) was calculated for quantitative analysis. This T/C metric reflects relative signal strength and was not used to infer absolute antibody titers in the absence of a validated calibration curve across heterogeneous clinical sera.

Statistical analysis was performed to evaluate diagnostic performance. A sample was classified as reference-positive when it tested positive by at least two *Brucella* antibody assays. Samples that did not meet this criterion were classified as reference-negative for anti-*Brucella* antibodies. Receiver operating characteristic (ROC) curve analysis assessed the overall assay performance. The correlation between ELISA and DFICT results was analyzed using GraphPad Prism. Qualitative agreement among ELISA, RBT, and DFICT tests was evaluated using kappa statistics.

## 3 Result

### 3.1 Preparation of *Brucella* LPS

LPS serves as the dominant surface antigen of Smooth *Brucella*, capable of strongly stimulating the host immune system and inducing high-titer specific antibody production. As one of the earliest antigens recognized during infection, anti-LPS antibodies emerge in the initial stages and persist throughout the disease course. Consequently, serological detection of LPS-specific antibodies provides a reliable early diagnostic indicator for Smooth brucellosis (Bedir et al., 2025; Lu et al., 2023).

**Figure 2:**
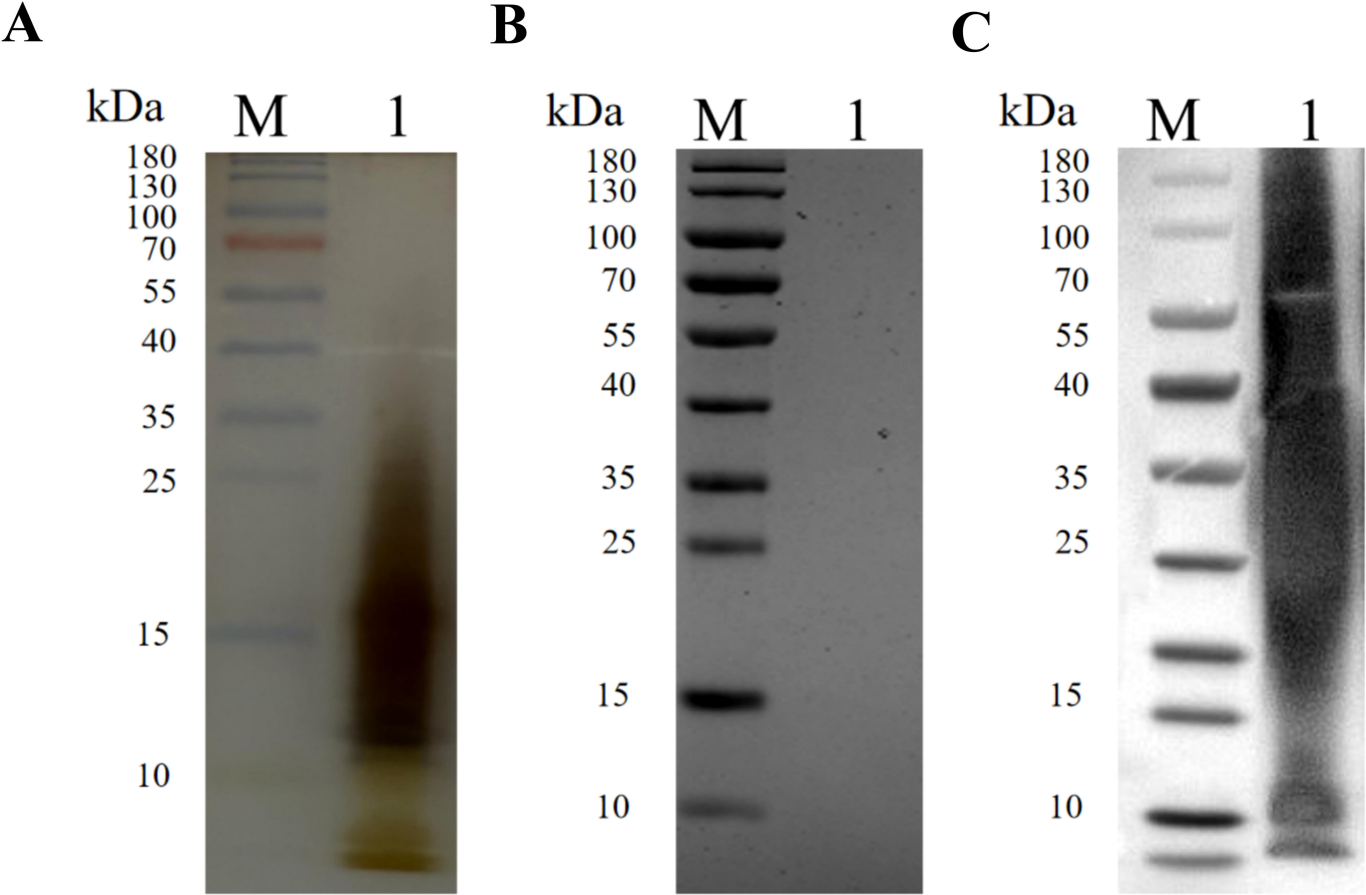
Analysis of the extracted LPS.**(A)** Silver staining, **(B)** Coomassie Brilliant Blue staining, and **(C)** Western blot. Lane 1: extracted LPS; Lane M: molecular weight markers.

In this study, we selected LPS as the capture antigen for DFICT development, where its structural integrity and purity critically determine the accuracy and reliability of subsequent assays. Silver staining results (**Figure 2A**) demonstrated uniformly dispersed bands, confirming successful extraction of LPS. To exclude protein contamination extracted LPS samples underwent SDS-PAGE separation followed by Coomassie Brilliant Blue staining, which revealed no detectable protein contamination (**Figure 2B**). Furthermore, western blot analysis verified that the extracted LPS maintained specific binding capacity against *Brucella* antibodies, substantiating its feasibility as a capture antigen (**Figure 2C**).

### 3.2 Preparation of gold-SPG conjugates

**Figure 3:**
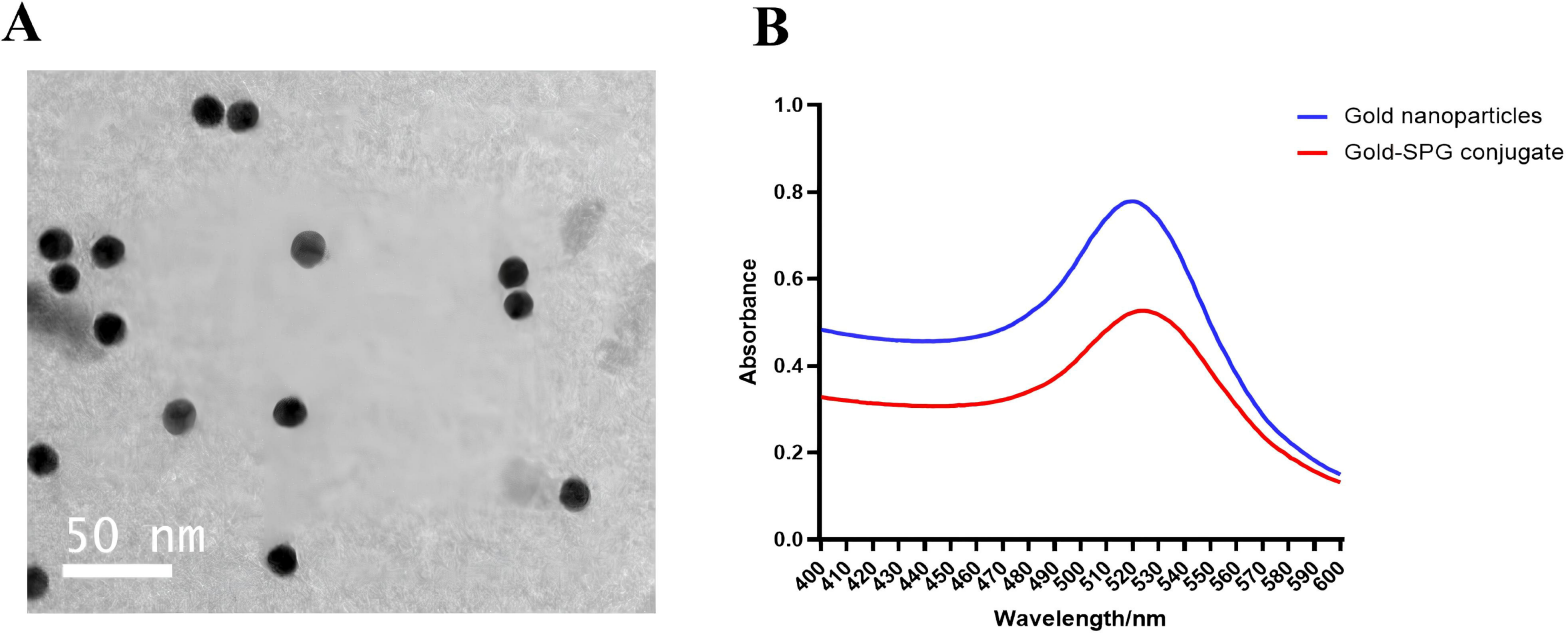
**(A)** TEM images of gold nanoparticles. **(B)** UV–vis spectra of the gold nanoparticle solution and the gold-SPG conjugate solution.

Colloidal gold particles were synthesized via trisodium citrate reduction of chloroauric acid. Primary characterization included transmission electron microscopy (TEM) revealing spherical, monodisperse particles with an average diameter of 12.7 nm (**Figure 3A**). UV-vis spectroscopy showed a characteristic absorption maximum at 520 nm for the pristine gold nanoparticles, which redshifted to 524 nm following SPG conjugation, confirming successful preparation of colloidal gold -SPG conjugates (**Figure 3B**). Compared with newer materials used in other ICT platforms, colloidal gold nanoparticles have well-established synthesis and conjugation protocols and are simple to operate. This feature further supports the DFICT system’s suitability for product development and field deployment (Huang et al., 2025; Tian et al., 2025).

### 3.3 Detection procedure and principle of the DFICT strip

The DFICT procedure initiates with applying 100μL of gold-SPG conjugate to reagent wells (R), followed by capillary-driven flow along the NC membrane. Subsequently, 5 μL of serum sample is added to sample wells (S), enabling migration and interaction with the gold-SPG conjugate (**Figure 1B**). Results are interpreted visually through color band development.

The underlying immunodetection principle (**Figure 1A**) involves specific anti-LPS antibodies in positive samples forming complexes with gold-SPG conjugates. These complexes migrate to the test line (T-line) where they bind immobilized LPS, generating a red band whose intensity correlates with antibody concentration. Unbound conjugates are captured at the control line (C-line) by SPG, producing a second red band. Negative samples lacking specific antibodies only develop the C-line band (**Figure 1B**). Absence of the C-line band invalidates the test regardless of T-line appearance.

### 3.4 Sensitivity of the DFICT strip

**Figure 4:**
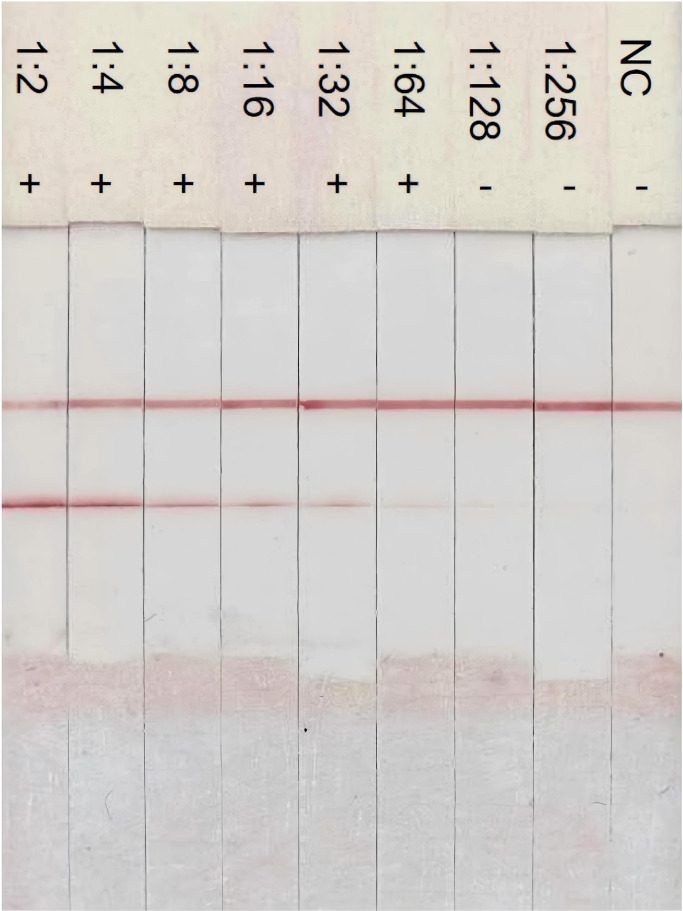
Sensitivity of the DFICT assay. A standard Brucella-positive sheep serum sample was serially diluted in 0.01 M PBS (pH 7.2) from 1:2 to 1:256 and tested with DFICT strips. Brucella-negative serum served as the negative control (NC). Experiments were performed in triplicate across three independent replicates. Similar results were observed with serially diluted bovine serum samples (data not shown).

The sensitivity of the DFICT test strips was evaluated by analyzing a series of dilutions of *Brucella*-positive standard serum from both sheep and bovine, with each dilution tested in triplicate. As illustrated in **Figure 4**, the color intensity of the T-line progressively diminished with increasing serum dilution. Notably, a clearly visible red T-line was still observed at a dilution of 1:64 for both species, demonstrating the assay’s ability to detect low concentrations of specific antibodies.

In conventional ICTs, sample volumes typically range from 10 μL to 100 μL (Ge et al., 2021; Wang et al., 2025). In contrast, the DFICT system achieves enhanced sensitivity with only 5 μL of serum through the implementation of a liquid gold conjugate as the detection reagent. This design ensures consistent and sufficient conjugate availability while promoting efficient migration to the test zone. The optimized flow dynamics minimize antibody loss during lateral flow, thereby significantly improving detection efficiency and reliability compared to traditional methods.

### 3.5 Specificity of the DFICT strip

**Figure 5:**
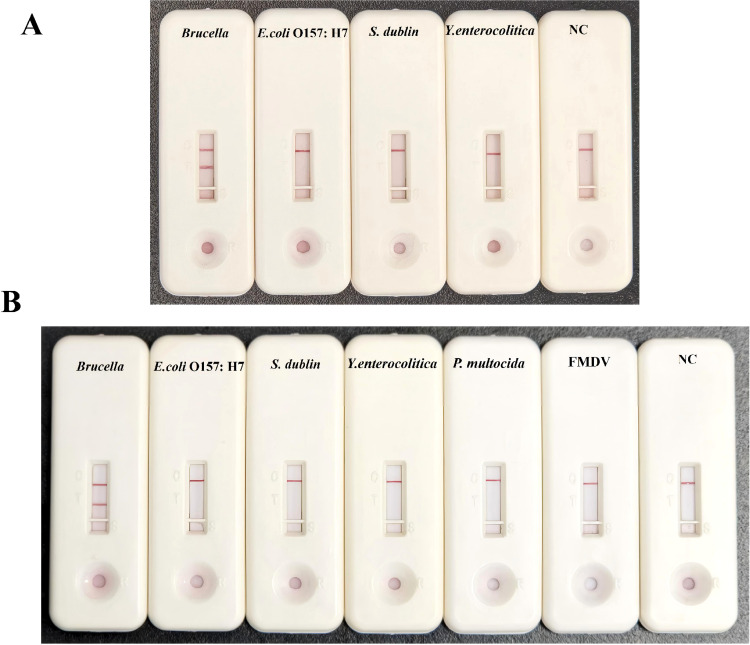
Specificity of the DFICT assay. **(A)** Testing of positive sheep sera against Brucella, E. coli O157: H7, S. dublin and Y. Enterocolitica using the DFICT. **(B)** Testing of positive bovine sera against Brucella, E. coli O157: H7, S. dublin, Y. enterocolitica, P. multocida and FMDV were tested using the DFICT. Negative sera was used as the negative control. The result patterns were consistent across repeated experiments.

To evaluate the specificity of the DFICT test strips, we tested serum samples from both sheep and bovine infected with *Brucella* and other common pathogens (*E. coli* O157, *Salmonella Dublin*, and *Yersinia enterocolitica*), as well as serum samples from bovine infected with Foot and Mouth Disease Virus (FMDV), and *Pasteurella multocida*. As shown in **Figures 5A, B**, revealed that only Brucella-positive sera produced distinct red bands at both test and control lines, while all sera from animals infected with non-Brucella pathogens tested negative. These results demonstrate that the DFICT strip exhibits high specificity for *Brucella* antibody detection without cross-reactivity to antibodies against other common ruminant pathogens.

### 3.6 Stability of the DFICT strip

Due to the potential need for large-scale field applications, simple storage methods and extended shelf life are of particular importance. The storage time and temperature significantly influence the effectiveness of the test strips (He et al., 2025; Shang et al., 2024). In our study, we found that when stored at 4 °C for 12 months, the DFICT strips maintained the same level of sensitivity as freshly prepared test strips, detected 1:64 diluted bovine or sheep-positive sera(**Supplementary Figure 1A**). When stored at RT, the DFICT strips retained their detection activity for *Brucella* for up to 6 months, but a reduction in sensitivity was observed after 9 months of continuous storage(**Supplementary Figure 1B**). Notably, the specificity of the test strips remained unchanged throughout the storage period, with no false-positive results recorded. Overall, the results indicate that the test strips have a validity of at least 12 months at 4 °C and 6 months at RT.

### 3.7 Diagnostic performance comparison

Brucellosis remains a significant zoonotic disease, for which bovine and sheep serve as the primary reservoirs and principal sources of human transmission (Vection et al., 2025). The RBT and ELISA are currently the most widely employed methods for detecting *Brucella* antibodies in domestic animals (Akar et al., 2025; Lu et al., 2024). In this study, we conducted parallel testing on 80 bovine and 161 sheep serum samples using RBT, iELISA, and cELISA to comprehensively evaluate the diagnostic accuracy and performance of DFICT strips.

**Figure 6:**
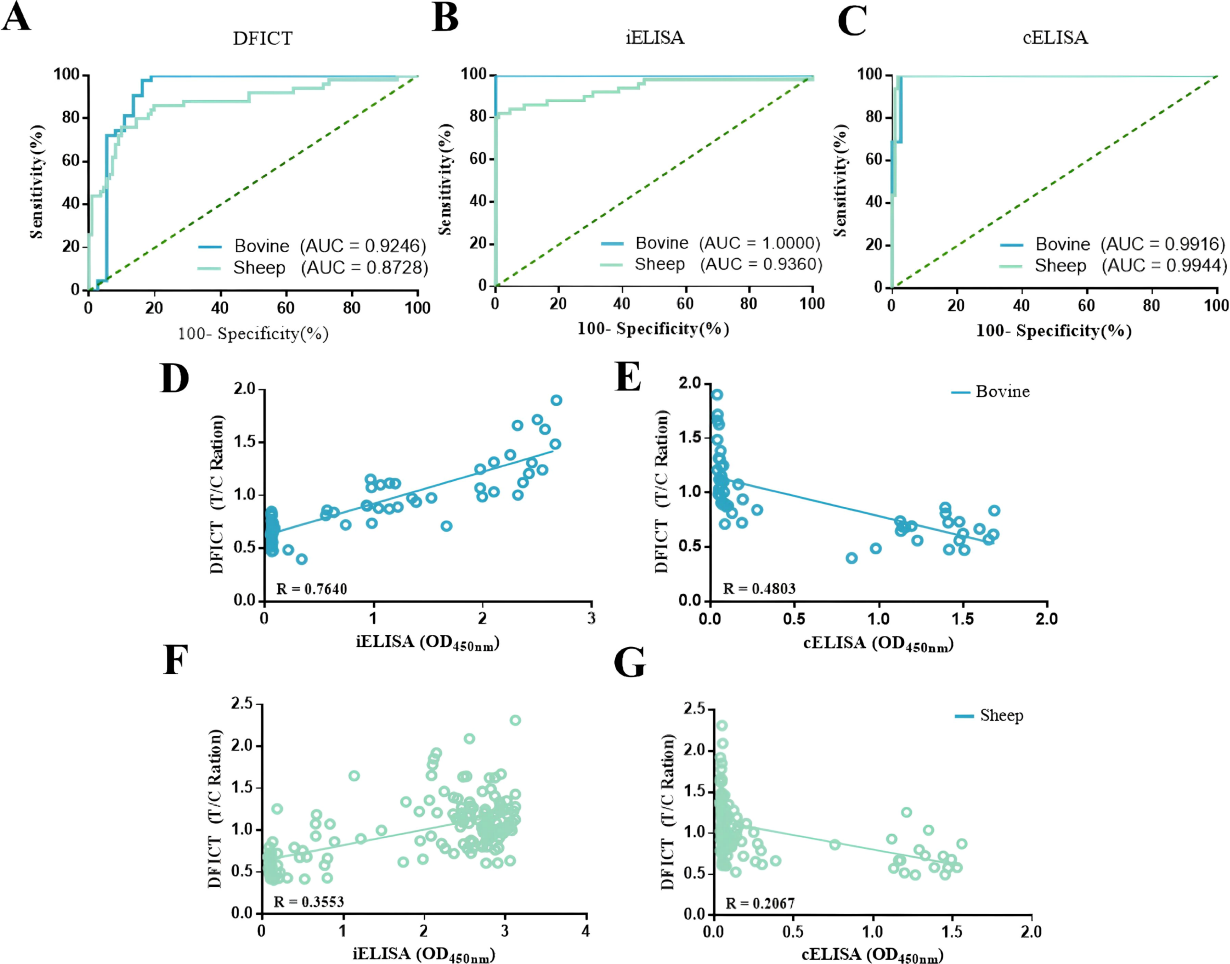
Diagnostic performance of the developed DFICT. **(A-C)** Receiver operating characteristic (ROC) analysis of DFICT **(A)**, iELISA **(B)**, and cELISA **(C)** for diagnosing brucellosis in bovine and sheep serum. **(D-G)** Correlation analysis between DFICT and iELISA in bovine **(D)** and sheep **(E)** serum, and between DFICT and cELISA in bovine **(F)** and sheep **(G)** serum.

The performance of DFICT test strips in bovine and sheep serum samples was examined. For bovine samples, DFICT demonstrated a diagnostic sensitivity of 94.6% and specificity of 100%. In sheep samples, sensitivity reached 98.2% while specificity was 84.0%. DFICT showed excellent agreement with conventional methods in bovine sera, with kappa values approaching 1.000 against RBT, cELISA, iELISA (**Table 1**). ROC curve analysis further confirmed strong diagnostic performance in bovine, yielding an AUC value of 0.9246, which compares favorably with iELISA (1.0000) and cELISA (0.9916) (**Figures 6A–C**).

**Table 1:** Comparison of DFICT with RBT, cELISA and iELISA results for sheep and bovine serum samples.

Group		RBT			Kappa	cELISA			Kappa	iELISA			Kappa
		+	-	Total		+	-	Total		+	-	Total	
Sheep	DFICT +	102	15	117	0.772	109	8	117	0.818	114	3	117	0.938
	DFICT -	1	43	44		4	40	44		1	43	44	
	Total	103	58	161		113	48	161		115	46	161	
Bovine	DFICT +	35	0	35	1.000	33	2	35	0.949	35	0	35	1.000
	DFICT -	0	45	45		0	45	45		0	45	45	
	Total	35	45	80		33	47	80		35	45	80	

A kappa statistic of ≥ 0.75 represents excellent agreement, 0.40 to 0.75 re-presents good to fair agreement, and < 0.40 represents poor agreement.

In sheep samples, among 117 DFICT-positive specimens, concordance rates with reference methods were 102 (RBT), 109 (cELISA), and 114 (iELISA). Of 44 DFICT-negative samples, 43 were negative by both RBT and iELISA, while 40 were negative by cELISA (**Table 1**). The AUC value for sheep sera was 0.8728 (**Figures 6A–C**), indicating good but comparatively reduced performance relative to bovine samples.

To further evaluate the concordance between DFICT and cELISA/iELISA, we implemented smartphone-based imaging combined with ImageJ (v1.53) to quantify DFICT signals through grayscale test-to-control (T/C) intensity ratio (Tabilin et al., 2024). Data analysis revealed a moderate correlation between DFICT and iELISA (r = 0.7640) in bovine serum, while a weaker association was observed with cELISA (r = 0.4803). In sheep samples, both correlations were substantially diminished, with obtained r-values of 0.3553 and 0.2067, respectively (**Figures 6D–G**). Supporting this hypothesis, RBT results demonstrated similar susceptibility to interference from serum contaminants like fibrinogen or cross-reactive antibodies, leading to diagnostic inaccuracies (Loubet et al., 2024). Consistent with this pattern, RBT exhibited reduced sensitivity (86.5%) and specificity (84.0%) in sheep samples compared to bovine samples. These collective findings suggest that the attenuated DFICT–ELISA correlations may arise from complex serum matrix effects that destabilize T- and C-line signal intensity and increase background. Future study will therefore focus on optimizing sample processing protocols and reagent formulations to enhance DFICT’s interference resistance.

## 4 Discussion

Brucellosis is a major zoonotic disease causing >2,000,000 human infections annually and threatening livestock production and public health. In animals, timely and accurate diagnosis is essential for early intervention and effective outbreak control. However, many current diagnostic approaches are limited by long turnaround times, dependence on specialized instrumentation, and susceptibility to cross-reactivity. Although bacterial isolation remains the confirmatory gold standard, routine surveillance and control programs increasingly rely on serological assays that are faster and sufficiently sensitive. Here, we developed a cost-effective dynamic-flow immunochromatographic test (DFICT) for rapid and accurate diagnosis of bovine and ovine brucellosis.

In this study, the DFICT architecture removes the serum-dilution step used in conventional brucellosis immunochromatographic assays, in which samples are buffered before loading into the sample well. Incorporating a liquid colloidal gold–SPG conjugate streamlines the workflow and improves user convenience (Prakash et al., 2021; Shome et al., 2025). Additionally, conventional ICT assays require the preparation of conjugate pads—a technically demanding process that relies on specialized equipment such as vacuum chamber or drying chambers (Zhao et al., 2024). In contrast, the DFICT system utilizes liquid gold conjugate reagents, eliminating the need for drying entirely. This innovation significantly streamlines the manufacturing process by removing requirements for drying equipment and strict humidity control during strip assembly, thereby increasing production throughput and reducing overall costs. We selected recombinant SPG for colloidal gold conjugation owing to its superior binding affinity for ruminant immunoglobulins compared to *Staphylococcal* protein A (SPA). Although both proteins interact with the Fc region of IgG across a broad range of species without interfering with antigen recognition, SPG exhibits stronger binding to antibodies derived from sheep and bovine. Moreover, SPG is capable of binding to human IgG as well as IgGs from other species such as mice, rats, rabbits, goats, and horses (Björck and Kronvall, 1984; Cheng et al., 2017). This broad reactivity profile suggests that the developed test strip could potentially be applied to brucellosis detection in humans and other animal species, though additional experimental validation would be necessary to confirm this cross-species utility.

Although cross-reactivity between smooth *Brucella* strains and other common bacteria such as *E. col*i O157 and S*. Dublin* have been reported in bovine (Perry and Bundle, 1990; Wu et al., 2024b), no such cross-reactivity was detected with the DFICT system. This enhanced specificity can be attributed to two main factors: the use of highly pure *Brucella* LPS obtained through a rigorous extraction process, and the optimized design of the DFICT platform, which improves analytical selectivity. In addition, the minimal serum volume required (5μL) reduces the potential introduction of cross-reactive substances, further minimizing interference risks.

Although smartphone-based quantification revealed some discrepancies in agreement between DFICT signals and iELISA/cELISA, the assay nevertheless showed good diagnostic concordance with the Rose Bengal plate agglutination test (RBT), indirect ELISA, and competitive ELISA. These findings indicate that DFICT provides a simpler and faster alternative. By using a liquid colloidal gold–SPG conjugate system, the technology eliminates drying steps, streamlines the workflow, shortens processing time, and minimizes the required serum volume. With a mature probe preparation process and high commercialization potential, results are visually interpretable within minutes, with the entire test taking approximately 10 minutes. Because of differences in antigen composition, assay format, and kit availability across rapid test strips, direct head-to-head comparison with other commercial or previously reported point-of-care strips was not performed in this study. Future work will include comparative evaluations of DFICT against other point-of-care rapid diagnostic methods to further validate overall performance across diverse use scenarios. Currently, the prevention and control of brucellosis in China typically employs a combined strategy of vaccination and isolation-purification. However, vaccine-induced antibodies can complicate routine serological surveillance and confirmation during monitoring and eradication efforts. Current commercial serological kits generally cannot reliably distinguish wild-type infection from vaccine-induced seropositivity or vaccine-associated infection, which may lead to misclassification and suboptimal control decisions. The DFICT developed here uses S-LPS from *Brucella melitensis* as the capture antigen and primarily detects anti–S-LPS antibodies. Consequently, it similarly struggles to differentiate between antibodies induced by vaccination and those arising from natural infection, constituting a limitation of this method in monitoring depopulation zones. DFICT is well suited for rapid, large-scale brucellosis screening, particularly in resource-limited settings and field conditions. Future work will prioritize developing smartphone-based quantitative analytics and mitigating matrix-interference effects to extend DFICT application within the One Health framework.

## Data Availability

The raw data supporting the conclusions of this article will be made available by the authors, without undue reservation.

## Supplementary material

Supplementary Figure 1 Storage stability of the DFICT strips under different conditions. **(A)** DFICT strips stored at 4 °C for 3, 6, 9, and 12 months retained the same sensitivity as freshly prepared strips, consistently detecting 1:64–diluted Brucella-positive bovine or sheep sera; NC, negative control serum. **(B)** DFICT strips stored at RT maintained detectable activity for up to 6 months, whereas a decrease in sensitivity was observed after 9 months of continuous storage; NC, negative control serum.
