# Supplementary figures and images for: Rapid serological detection of smooth Brucella strain antibodies in bovine and sheep using a dynamic flow immunochromatographic test
Source: Front Cell Infect Microbiol. 2026 Jun 4;16:1804374. doi: 10.3389/fcimb.2026.1804374 (PMC13275397; doi:10.3389/fcimb.2026.1804374)

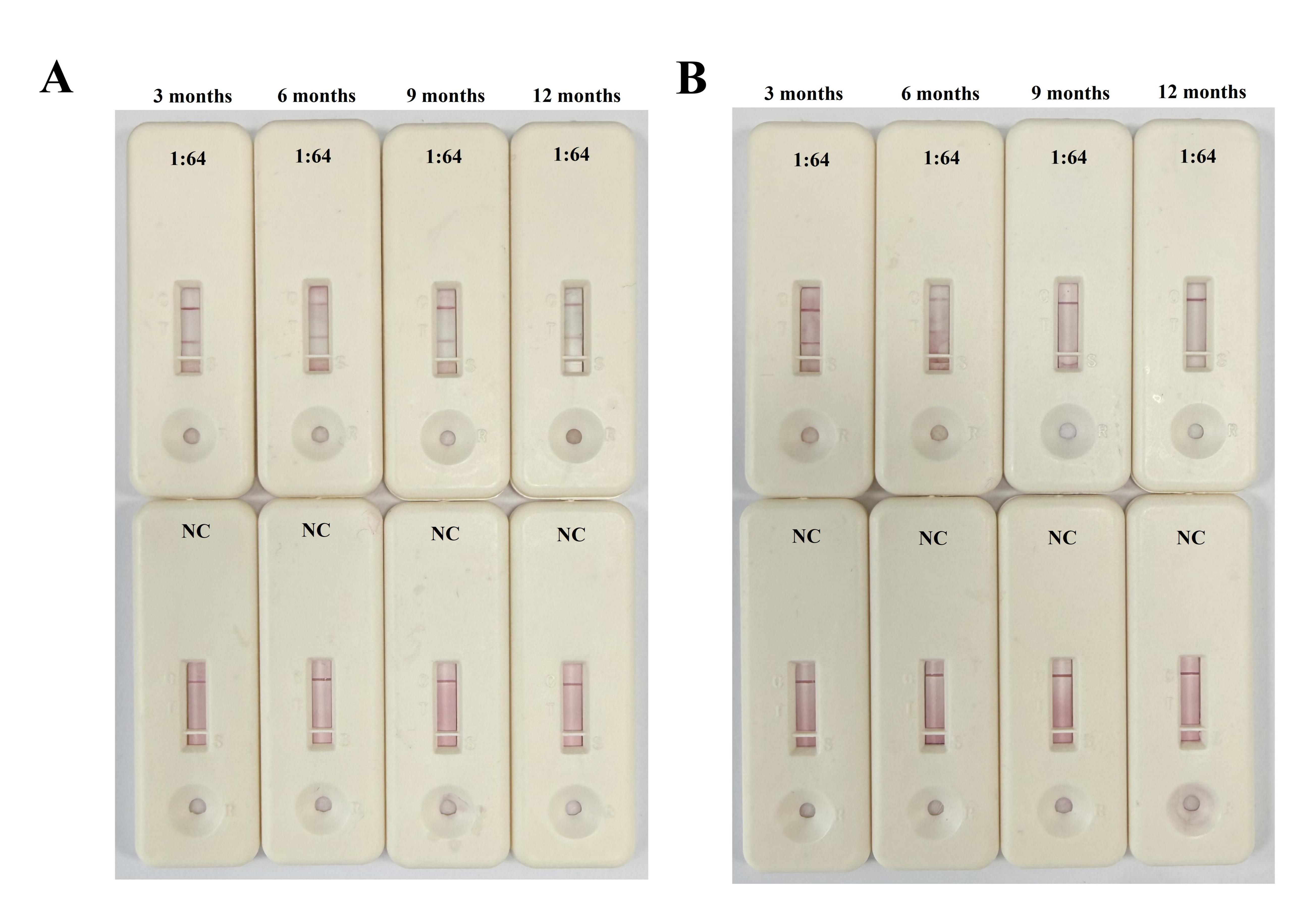

Supplement: Supplementary Figure 1 — Storage stability of the DFICT strips under different conditions. (A) DFICT strips stored at 4 °C for 3, 6, 9, and 12 months retained the same sensitivity as freshly prepared strips, consistently detecting 1:64–diluted Brucella-positive bovine or sheep sera; NC, negative control serum. (B) DFICT strips stored at RT maintained detectable activity for up to 6 months, whereas a decrease in sensitivity was observed after 9 months of continuous storage; NC, negative control serum. [file Image1.jpeg]
